# The neural basis of explicit and implicit emotion regulation: a coordinate-based meta-analysis

**DOI:** 10.1093/oons/kvag013

**Published:** 2026-09-18

**Authors:** Yanyao Zhou, Vickie T Y Lee, Dorita H F Chang, Charlene L M Lam

**Affiliations:** Laboratory of Clinical Psychology and Affective Neuroscience, The University of Hong Kong, Hong Kong, China; Laboratory of Clinical Psychology and Affective Neuroscience, The University of Hong Kong, Hong Kong, China; Brain and Behavior Laboratory, The University of Hong Kong, Hong Kong, China; Laboratory of Clinical Psychology and Affective Neuroscience, The University of Hong Kong, Hong Kong, China

**Keywords:** explicit, implicit, emotion regulation, reappraisal, extinction, activation likelihood estimation

## Abstract

Emotion regulation (ER) is a transdiagnostic factor in psychopathology. However, research heavily dominates explicit emotion regulation, leaving implicit mechanisms poorly understood. We investigated whether explicit and implicit emotion regulation, conceptually differentiated by the explicitness of their regulatory goals, exhibit distinct neural signatures. We conducted a coordinate-based activation-likelihood estimation meta-analysis comprising 79 explicit (2961 subjects) and 29 implicit (1329 subjects) ER experiments among healthy subjects, utilizing a rigorous leave-one-out (LOO) sensitivity analysis to ensure spatial robustness. Explicit ER engaged left-lateralized frontoparietal network, robustly activating the inferior, superior, and middle frontal gyri, as well as the middle and superior temporal gyri. In contrast, implicit ER recruited a bilateral network involving subcortical integration hubs and emotional reactivity areas, specifically the insula, cingulate gyrus, caudate, and lentiform nucleus. Conjunction analyses revealed that shared neural engagement was limited primarily to the bilateral insula and right inferior frontal gyrus. Explicit ER recruits regions responsible for goal-directed behaviors while implicit ER targets regions related to emotion and affect processing. The difference between implicit and explicit emotion regulation may lie in the level of consciousness involved. This suggests that clinical interventions could be optimized by targeting these disparate networks.

## Introduction

Emotion regulation (ER) is broadly defined as the process by which people influence their expressions and experience of emotions (McRae and Gross 2020). It is closely linked with our mental health (Gratz et al. 2015, Webb et al. 2012), and has long been recognized as a transdiagnostic factor in psychopathology (Aldao et al. 2010, 2016, Lincoln et al. 2022, Sloan et al. 2017). Maladaptive ER is widely reported in various psychiatric disorders such as depression (Joormann and Stanton 2016, Vanderlind et al. 2020), anxiety disorders (Helbig-Lang et al. 2015), post-traumatic stress disorder (McLean and Foa 2017) and eating disorders (Brockmeyer et al. 2014). Moreover, many clinical interventions of mental disorders, such as cognitive behavioral therapy (CBT) and dialectical behavioral therapy, also target ER as one of the cores in therapeutic sessions (Goldin et al. 2014, Harvey et al. 2019, Lincoln et al. 2022, Rozakou-Soumalia et al. 2021, Spinhoven et al. 2018).

The significance of ER in psychopathology and mental health has motivated a large number of research to study its mechanisms and effects on general and clinical population. Theoretically, ER is a process that can occur at any temporal stage of a series of interconnected cycle of emotion generation (Gross 2015a, 2015b, Gross and Diener 1998, Lincoln et al. 2022). ER has its own physiological, behavioral and cognitive feature (Bridges et al. 2004, Rottenberg and Gross 2007). Empirically, most laboratory-based tasks of ER focus on the deliberate use of ER strategies (Saraiya and Walsh 2015). Similarly, most measures of ER quantify the deliberate use of ER strategies (Aldao et al. 2010), which refer to the methods individuals use to modulate the experience or expression of their emotions (Martínez-Priego et al. 2024). Popular measures include the Emotion Regulation Questionnaire (Gross and John 2003) and short or full form of the Difficulties in Emotion Regulation Scale (Bjureberg et al. 2016, Gratz and Roemer 2004).

A major limitation of both widely used tasks and measures of ER is that ER is narrowly considered as a deliberate process where people consciously reflect, report and execute on different aspects of ER. Nevertheless, ER can happen without people’s deliberate effort (Koole et al. 2015, Mauss et al. 2007, Saraiya and Walsh 2015), and ER strategies can also differ in the amount of conscious management of emotional responses involved (Gyurak et al. 2011, Mauss et al. 2007). Following this definition, ER strategies can be classified on a continuum that differs on the level of conscious and deliberate regulation involved. Implicit and explicit ER are thus on two opposite ends of this spectrum.

The field currently has mixed definitions regarding implicit and explicit ER. Some define implicitness as the absence of consciousness and ER goals, while others regard it as the automaticity of regulatory control (Braunstein et al. 2017). Nevertheless, consciousness and effort are rather two different constructs (Shepherd 2023). To avoid using the constructs of consciousness and effort interchangeably, we followed Braunstein et al.’s (2017) definitions of implicitness and explicitness. According to their definitions, ER is measured on two orthogonal dimensions: the explicitness of ER goal (implicit vs. explicit) and the controllability of the ER process (controlled vs. automatic). We thus refer implicitness as the absence of ER goal only, regardless of the effort needed during the regulatory process.

Surprisingly, research on implicit ER is relatively limited. A thorough study of implicit ER is necessary because maladaptive implicit ER has been suggested to cause psychiatric disorders (Bruce et al. 2013, Gyurak et al. 2011) and deficits in implicit ER are widely reported among patients with prevalent psychological disorders such as mood and anxiety disorders (Dryman and Heimberg 2018, Joyal et al. 2019, Picó-Pérez et al. 2017, Schäfer et al. 2017, Wang et al. 2024, Wright et al. 2014).

To differentiate between implicit and explicit ER and to unravel their neural basis, this study sought to examine whether they are also on a continuum at the neural level, as they are suggested to be at a conceptual level. We wonder if these two types of ER recruit similar brain regions, but to different extents during the regulatory process. Investigating the neurological foundation of these two types of ER, as in other neuroscientific studies, would advance the understanding of the mechanisms involved (Yen et al. 2023). Furthermore, understanding the relationship between implicit and explicit ER could lead to the development of new therapeutic interventions, which leverage the reciprocal influence of these two types of ER, a concept that is still under debate based on mixed evidence (Fu et al. 2008, Kitamura et al. 2022, Moser et al. 2010, Pan et al. 2022, Vazquez et al. 2018, Yang and Pourtois 2022).

Prior studies have only targeted a limited number of ER strategies (Frank et al. 2014) and primarily focused on reappraisal for explicit ER and extinction learning for implicit ER (Picó-Pérez et al. 2019). Across populations, hubs such as the cingulate cortex and frontal regions seem to function as the common regulatory hubs for ER. Specifically, in healthy populations, foundational meta-analyses of explicit ER demonstrate consistent recruitment of a prefronto-parietal cognitive control network, alongside the deactivation of limbic regions (Buhle et al. 2014, Diekhof et al. 2011, Ochsner et al. 2012). Conversely, studies mapping implicit ER in healthy individuals suggest the recruitment of prefrontal regions such as rostral anterior cingulate cortex (Fitzgerald et al. 2020), ventrolateral prefrontal cortex (Tupak et al. 2014) and regions that have rich connections to subcortical areas like insular cortices (Fullana et al. 2018). Among clinical populations, frontal areas – in particular, the anterior middle cingulate cortex and (pre-)supplementary motor area – alongside the superior temporal gyrus and angular gyrus serve as the key neural substrates for explicit ER (Kohn et al. 2014). Regarding implicit ER, hypoactivation in the right medial frontal and anterior cingulate gyri, as well as the left middle and superior temporal gyri and hyperactivation in the left medial, superior, and middle frontal gyri were found among patients with mood and anxiety disorders (Dalton et al. 2025).

While hubs such as the cingulate cortex and diverse frontal regions frequently emerge across studies, it remains unclear whether these distinct ER modalities fall on a shared neural continuum. Therefore, the core question of this study was whether these previously identified regions remain robust when moving beyond narrow definitions to evaluate a comprehensive spectrum of regulatory strategies. To address this, we conducted a coordinate-based neuroimaging meta-analysis using activation likelihood estimation (ALE) to examine the similarities and differences in the neural bases of explicit and implicit ER. While we initially targeted studies focusing on both healthy subjects and patients with mood and/or anxiety disorders – given their prevalence in ER research (Aldao et al. 2010) – our final analyses focused solely on healthy populations due to insufficient sample sizes in the clinical groups. Ultimately, by directly contrasting different ER paradigms, we aimed to clarify how the explicitness of the regulatory goal drives neural recruitment.

Because explicit ER strategies involve an active regulatory goal, we hypothesized they would activate more regions associated with goal-directed behavior and cognitive control, such as the prefrontal cortex (Mannella et al. 2013). Conversely, we expected implicit ER to more strongly activate subcortical regions responsible for emotional responses, such as the thalamus (Barson et al. 2020), as well as regions for emotional inhibition like the ventrolateral prefrontal cortex (Hung et al. 2018), while relying less on broad cognitive control hubs, such as the DLPFC and dorsal anterior cingulate cortex (E. H. Smith et al. 2019). Finally, we hypothesized that both ER types would demonstrate functional overlap in emotion generation areas (e.g. the amygdala; Pichon et al. 2012) and awareness-modulating hubs (e.g. the insula; R. Smith and Lane 2015; R. Zhang et al. 2017; Zhuang et al. 2021).

## Method and materials

### Search strategy

We pre-registered our study on PROSPERO (Zhou et al. 2024; CRD42024505144). A literature search was conducted in three databases: PubMed, Web of Science, and Embase. We searched for the literature of explicit and implicit ER using the combinations of the following keywords: ‘emotion regulation’; ‘reappraisal’; ‘distraction’; ‘suppression’; ‘extinction’; ‘fear conditioning’; ‘affect labelling’; ‘emotional stroop’; ‘emotional go/no-go’; ‘psychological placebo’. We additionally targeted imaging literature using the combination of keywords: ‘functional magnetic resonance imaging’; ‘positron emission tomography’; ‘fMRI’; ‘PET’.

We limited our search to the period between January 1, 2014, and June 21, 2026, to ensure the inclusion of studies with more stable and standardized neuroimaging quality, which improved significantly after the late 2000s (Kabasawa 2022). Additional literature was identified from previous review articles and meta-analyses to check for possible omissions in our original search and to ensure the comprehensiveness of the coverage.

### Inclusion and exclusion criteria

Following the framework established by Braunstein et al. (2017), we conceptually distinguished between explicit ER - which involves an explicit goal to regulate emotion using specific strategies - and implicit ER, which occurs without an explicit regulatory goal. Because implicit ER does not involve deliberate strategies, it is instead operationalized using experimental paradigms that incidentally require ER, for the purpose of task performance. Articles were included in this meta-analysis if they met the following criteria:

1) Included adult participants (aged 18 years or older) who were either healthy or diagnosed with a mood and/or anxiety disorder (e.g. depression, anxiety disorders, PTSD). Articles were excluded if the clinical group had other comorbid psychiatric or neurological disorders. Studies containing mixed adult and adolescent samples were excluded unless a separate analysis was reported for the adult group.
2) Examined an eligible explicit ER strategy (reappraisal, suppression, distraction, selective attention), the explicit ER process of psychological placebo, or an eligible implicit ER paradigm (extinction, affect labeling, emotional go/no-go, or the emotional Stroop task) within the target population.
3) Conducted whole-brain imaging and whole-brain analyses, and reported peak coordinates for brain activations in a standard anatomical reference space (e.g. Talairach or Montreal Neurological Institute [MNI]). Studies that performed only region-of-interest (ROI) analyses were excluded from this meta-analysis. Coordinates reported in Talairach space were converted to MNI space using the coordinate conversion tool in GingerALE 3.0.2 (Eickhoff et al. 2009, Turkeltaub et al. 2012).

Our inclusion criteria for explicit ER were highly comprehensive, capturing the vast majority of the established literature. Regarding implicit ER, our approach would lead us to omit several paradigms sometimes characterized as implicit regulation – such as implicit cognitive reappraisal (Shahane et al. 2019, Wang et al. 2017) and the Shifted-Attention Emotion Appraisal Task (Anderson et al. 2003). Although omitting these means some potentially relevant studies were not analyzed, these paradigms lack standardized operational definitions and are not widely utilized in the broader implicit ER field. For instance, the operationalization of implicit cognitive reappraisal exhibits high methodological heterogeneity: some studies quantify it by measuring spontaneous neural pattern correspondence during passive viewing without explicit instruction (e.g. Shahane et al. 2019), while others utilize semantic priming – presenting descriptive words prior to emotional stimuli to unconsciously alter the appraisal process (e.g. Wang et al. 2017). Consequently, we adhered strictly to the target paradigms defined by Braunstein et al. (2017) to maintain methodological consistency by focusing on paradigms that are well-established and theoretically grounded.

Two reviewers (Y.Z., V.L.) independently performed the screening and made inclusion decisions based on the established criteria. In cases of disagreement, the two reviewers discussed the discrepancy to reach a consensus. If an impasse occurred, a third reviewer (C.L.) was consulted. The two reviewers further independently extracted relevant information, including: coordinates, T/Z scores, type and number of studied populations, type of intervention, neuroimaging reference template, neuroimaging analysis software, type of stimuli, ER strategy/task, author, year, and DOI. A third reviewer (T.R. or C.A.) independently verified the accuracy of all recorded information. Authors were contacted if the reported results contained ambiguous information, such as unclear T-thresholds used for the whole-brain analysis or missing T/Z scores for reported coordinates. Papers were excluded from the final analysis if no clarification was obtained from the authors.

### Classification of explicit and implicit emotion regulation

To classify ER strategies/tasks into explicit and implicit categories, we followed the definitions proposed by Braunstein et al. (2017). We focused solely on the dimension of the explicitness of the regulatory goal because consciousness of the regulatory process cannot be deliberately trained, making it less applicable to interventional research. Nevertheless, the goal of ER is trainable (Denny 2020), and the ER goal is closely linked with mental health (Brandão et al. 2023). According to Braunstein et al. (2017), explicit ER strategies have a clear and deliberate goal of ER. In contrast, implicit ER strategies refer to tasks that do not prioritize ER as the explicit goal of the strategy/tasks. We therefore classified cognitive reappraisal, suppression, distraction, selective attention, and psychological placebo as explicit ER strategies, and classified extinction, affect labeling, emotional go/no-go, and the emotional Stroop task as paradigms that involve implicit ER processes.

To ensure that the extracted peak coordinates from each study accurately reflected the neural correlates of the target ER type, we extracted results from specific fMRI contrasts. We first extracted coordinates involved in ER in general using the contrast *Implicit/Explicit ER > Non-regulation*. Positive and negative stimuli used in target experimental conditions were not analyzed separately due to insufficient sample size, though data for distinctively valenced conditions were extracted separately. Sample eligible fMRI contrasts for the explicit ER included: *regulate negative > watch negative* (reappraisal), *distance > permit* (reappraisal), *suppress negative > feel negative* (suppression), *distraction > control* (distraction), *unpleasant arousing focus > unpleasant no focus* (selective attention) and *placebo > control during pain induction* (psychological placebo). For implicit ER, sample eligible contrasts included: *Conditioned Stimulus (CS)+ > CS-* (extinction), *incongruent > congruent* (emotional Stroop), *disgust no-go > neutral no-go* (emotional go/no-go), and *affect label > gender label* (affect labeling).

The distinct regions involved in explicit and implicit ER were identified by contrasting the ALE outputs of *Explicit ER > Non-regulation* and *Implicit ER > Non-regulation*. Shared regions were identified via a conjunction analysis of these two contrasts.

### Analysis: Activation-likelihood estimation (ALE)

We performed ALE (Eickhoff et al. 2009, 2012, Turkeltaub et al. 2012) to identify overlapping clusters of activations (convergence) across experiments that tested explicit ER and implicit ER (conjunction analysis), as well as differences in clusters that were activated uniquely by each type of ER (divergence).

We used GingerALE 3.0.2 to perform this coordinate-based meta-analysis. This algorithm models the coordinates extracted from each experiment as a three-dimensional Gaussian distribution centered on the reported foci (peaks). The algorithm scales the Gaussian probability distribution according to the study’s sample size to account for spatial uncertainty. By computing the union of activation probabilities for each voxel across studies, an ALE map is created. This map is then tested against the null hypothesis that the experiment-specific foci spread uniformly over the brain, rather than clustering at specific regions. Each region is then associated with a *p*-value indicating the significance of its contribution to the target ER condition.

We tested for the convergence of brain activations for explicit and implicit ER conditions. Additionally, we performed contrast analyses to identify regions specific to explicit ER (relative to implicit ER), and vice versa. Analyses of convergence and divergence were only performed if at least 17 studies were available for each type of ER. As recommended by Eickhoff et al. (2016), this minimum threshold is necessary when applying cluster-level family-wise error (FWE) correction to prevent individual studies from disproportionately driving the results; specifically, it ensures that a single dominant experiment contributes less than 50% to a given cluster, and the two most dominant experiments combined contribute less than 80%. For the convergence analysis, we corrected for multiple comparisons with an FWE rate of 0.05 at the cluster level, using a cluster-forming threshold of *p* < 0.001. We performed 2000 permutations for each convergence map, meaning the algorithm randomly relocated the foci across the brain 2000 times. The choice of the FWE and cluster-forming thresholds aligned with the recommendations of Eickhoff et al. (2016). For the contrast analysis, we set the *p*-threshold at 0.05 and the minimum cluster size to 200 mm^3^. We used the Oxford Center for Functional Magnetic Resonance Imaging of the Brain Software Library (FSL) to visualize the result of coordinate-based meta-analysis.

### Quality assessment and sensitivity analysis: Leave-one-out (LOO) spatial robustness

To evaluate the robustness of the ALE findings and ensure that the identified neural regions were not disproportionately driven by any single study, we conducted a comprehensive leave-one-out (LOO) sensitivity analysis as recommended by guidelines of neuroimaging meta-analysis (Müller et al. 2018). This approach iteratively excluded one study at a time from both the explicit and implicit datasets. The coordinate-based meta-analysis was recomputed for every newly generated LOO dataset across all iterations. LOO analysis was conducted using the NiMARE v.0.20.0 package (Salo et al. 2026) in Python 3.11. We did not opt for traditional Risk of Bias assessment tools because they are designed for clinical trials and epidemiological studies and do not capture the variations in coordinate-based meta-analysis precisely, which arises from idiosyncratic study effects – where a single experiment reporting numerous foci or using lenient statistical thresholds disproportionately drives the group-level spatial convergence.

For each LOO iteration, we applied rigorous statistical thresholding to match the metrics used in our main analysis conducted with GingerALE. Specifically, for the primary group maps, we applied a FWE cluster-level correction utilizing 2000 Monte Carlo permutations with a voxel-forming threshold of *p* < 0.001. Conjunction analyses between the explicit and implicit maps were computed using minimum statistic logic across the FWE-corrected main effects, with a minimum cluster size of 200mm^3^ (Eickhoff et al. 2012). To balance statistical rigor and computational cost, we performed 2000 permutations for each contrast (*Explicit ER > Implicit ER and Implicit ER > Explicit ER*) and 2000 permutations utilizing ALE subtraction in NiMARE.

To systematically evaluate the robustness of our LOO sensitivity analysis, we computed the overlap rate, Dice similarity coefficient (DSC), survival rate, and identified the most disruptive study for each cluster. Higher overlap and survival rates reflect a neural region’s high resilience to dataset variations, higher DSC values (ranging from 0 to 1) reflect more precise spatial alignment between the LOO and baseline clusters. The DSC is a standard metric for quantifying spatial reproducibility in neuroimaging and is monotonically related to the Jaccard index, which is frequently recommended and used by prior literature for assessing spatial reliability (Crum et al. 2006, Liu et al. 2023). A baseline cluster was defined as ‘strictly surviving’ a given iteration if it simultaneously met three criteria: 1) structural viability with a minimum significant cluster size of 25 voxels (200 mm^3^); 2) spatial reproduction demonstrating at least 10% coverage of the baseline cluster; and 3) peak stability, defined as a peak location drifting no more than 15 mm from the baseline peak, which accounts for the standard spatial smoothing kernels applied in coordinate-based meta-analyses (Radua and Mataix-Cols 2009). For contrast analyses, significance was further constrained by a threshold of *Z* ≥ 1.645 (equivalent to a one-tailed *p*-threshold of 0.05) and masked by the respective main effects. This multifaceted evaluation ensures that our identified regions reflect reliable neural recruitment patterns rather than idiosyncratic study effects.

## Results

### Study inclusion

Our search returned 11 262 abstracts (Fig. 1). All screenings were conducted in EndNote (Gotschall 2021). After duplicates removal, we started with 6096 abstracts, which further yielded in 1002 articles for full-text screening. The final inclusion contained 100 articles, among which 73 studies studied at least one of the target explicit ER strategies, 26 studied at least one of the target implicit ER strategies, and one study studied both implicit and explicit ER. The most frequently researched explicit ER strategy was reappraisal, followed by suppression and distraction. The most extensively studied implicit ER strategy was extinction, followed by emotional go/no-go and emotional stroop. The characteristics of the included studies are shown in Table 1 and Table 2.

**Figure 1:**
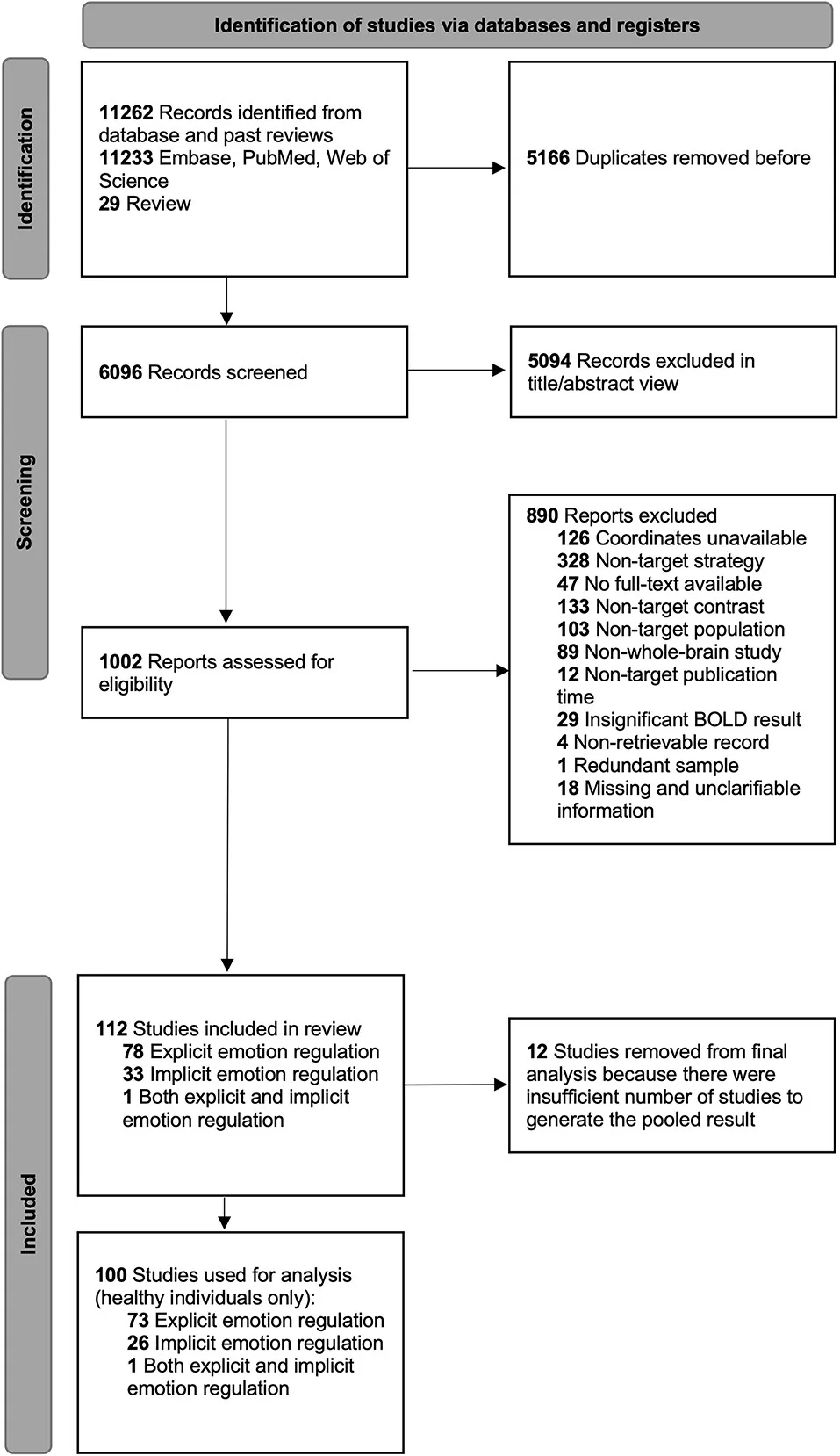
PRISMA flowchart of study inclusion and exclusion.

**Table 1:** Summary of Implicit Emotion Regulation Studies Meeting the Inclusion Criteria

**Authors**	**Task**	**Type of Emotion Regulation**	**Nhealthy (%Female)**	**Ndisease (%Female)**	**AgeHealthy** ^**a**^ **M (SD)**	**AgeDisease** ^**a**^ **M (SD)**	**Intervention**	**Stimuli**
Alders et al., 2019	Emotional Stroop	Controlled	30 (73%)	48 (69%)	33.2 (9.8)	34.7 (12.2)	NA	Emotional faces (Ekman & Friesen)
Alders et al., 2020	Emotional Stroop	Controlled	30 (73%)	42 (66.7%)	33 (10)	34.62 (NA)^b^	NA	Emotional faces (Ekman & Friesen)
Berlin et al., 2015	Emotional Go/No-Go	Controlled	10 (50%)	9 (44.4%)	38.70 (11.97)	38.33 (11.23)	NA	Emotional images (IAPS)
Burklund et al., 2015	Affect Labelling	Controlled	15 (60%)	67 (47.8%)	27.94 (6.8)	28.35 (7.25)	NA	Emotional faces (NimStim)
Cha et al., 2021	Emotional Go/No-Go	Controlled	29 (62%)	89 (73%)	24.8 (3.4)	24.4 (NA) ^b^	NA	Emotional faces (Unknown)
Chechko et al., 2014	Emotional Stroop	Controlled	24 (50%)		28.7 (2.9)		NA	Emotional faces (FEBA)
de voogd et al., 2018	Extinction	Automatic	24 (50%)		24.8 (3.6)		EMDR	US: Electrical shock CS: Shapes
Dunsmoor et al., 2019	Extinction	Automatic	46 (63%)		23.43 (4.76)		NA	US: Electrical shock CS: Faces (Ekman & Friesen) NFE: Sound
Ebrahimi et al., 2017	Extinction	Automatic	32 (50%)		27(1)		D-cycloserine / Placebo	US: Images of 2€ coin with plus or minus sign CS: Shapes
Ewald et al., 2014	Extinction	Automatic	26 (65.4%)		23.3 (NA) ^b^		NA	US: Electrical shock CS: Colored lights
Fang et al., 2022	Emotional Stroop	Controlled	20 (75%)	18 (77.8%)	29.5 (8.59)	32.11 (10.77)	NA	Colored words
Faul et al., 2020	Extinction	Automatic	40 (45%)		24.55 (4.16)		NA	US: Electrical shock CS: Male avatar
*Fitzgerald et al., 2020	Emotional Stroop	Controlled	49 (67%)		25.24 (7.98)		NA	Letters on emotional faces (Ekman & Friesen)
Fournier et al., 2017	Emotional Stroop	Controlled	28 (60.71%)	135 (67.41%)	37.39 (16.08)	36.51 (12.56)	NA	Emotional faces (Unknown)
Hagedorn et al., 2021	Extinction	Automatic	60 (50%)		24.5 (3.8)		Cortisol / Placebo	US: Electrical shock CS: Shapes
Hagedorn et al., 2022	Extinction	Automatic	60 (50%)		23.9 (4.1)		Hydrocortisone (cortisol) / Placebo	US: Electrical shock CS: Shapes
Hassel et al., 2020	Emotional Stroop	Controlled	36 (72.2%)		33 (9.9)		NA	Red uppercase lettering on emotional faces (Ekman & Friesen)
Hennings et al., 2020	Extinction	Automatic	48 (66.7%)		23.5 (NA)^b^		NA	US: Electrical shock CS: Images of animals and tools (lifeonwhite.com)
Huang et al., 2019	Emotional Stroop	Controlled	40 (62.5%)	55 (69.1%)	68.1 (5.3)	66.36 (5.42)	NA	Neutral or emotional words
Keller et al., 2022b	Extinction	Automatic	23 (60.9%)		23.69 (5.63)		NA	US: Electrical shock CS: Images of animals, tools, and food
Kirlic et al., 2022	Extinction	Automatic	52 (NA)	230 (NA)	31.98 (11.37)	35.65 (11.08)	NA	US: Scream sound CS: Neutral images
Kroes et al., 2016	Extinction	Automatic	46 (58.7%)		21.68 (NA)^b^		Propranolol / Placebo	US: Electric shock CS: Images of colored lamps
Lin et al., 2026	Affect labelling	Controlled	37 (45.9%)		20.89 (2.33)		NA	Emotional images (EmoPics & IAPS)
*Loeffler et al., 2019	Emotional Stroop	Controlled	26 (53.8%)	26 (53.8%)	35.31 (11.23)	35.27 (11.03)	NA	Red words on emotional faces (FACES)
Michel et al., 2024	Emotional Stroop	Controlled	37 (89%)		48.38 (11.43)		NA	Colored words with deceased or living attachment
Miedl et al., 2020	Extinction	Automatic	58 (100%)		22.8 (4.1)		NA	US: Aversive film clips CS: Neutral faces
Molapour et al., 2015	Extinction	Automatic	20 (50%)		22.39 (3.82)		NA	US: Electrical shock CS: White and Black American males neutral faces
Morr et al., 2022	Extinction	Automatic	82 (46.3%)		26.39 (5.83)		NA	US: Electrical shock CS: Social and non-social images
Morriss et al., 2015	Extinction	Automatic	22 (54.5%)		23.59 (2.75)		NA	US: Female scream sound CS: Colored squares
Schwarzmeier et al., 2017	Extinction	Automatic	10 (70%)	10 (70%)	27.6 (8.0)	27.5 (8.5)	NA	CS: Neutral male faces (Ekman & Friesen) US: Aversive panic scream sound
Seo, J. et al., 2022	Extinction	Automatic	63 (57.1%)	63 (79.4%)	23.54 (4.56)	24.79 (5.55)	NA	US: Electrical shocks CS: Colored lights
Seo et al., 2026	Extinction	Automatic		30 (93%)	24.63 (4.09)^b^		NA	US: Electrical shocks CS: Colored lamps
Stickel et al., 2019	Emotional Stroop	Controlled	76 (100%)		30.78 (4.69)		NA	Emotional faces (FEBA)
Visser et al., 2021	Extinction	Automatic	79 (67.1%)		22.09 (NA)^b^		NA	US: Electrical shock CS: Neutral faces and image of house
Wroblewski et al., 2022	Extinction	Automatic	93 (45.16%)		32.93 (10.5)		NA	US: Electrotactile stimulus CS: Neutral male face
Zhuang et al., 2021	Emotional Go/No-Go	Controlled	227(50.67%)		21.62 (2.32)		NA	Emotional words

*Note.* This table contains all studies that met the inclusion criteria. Not all studies were further used in the analysis due to insufficient sample size for some contrasts (e.g., Healthy Control vs. Patient for ER). Abbreviations: CS = Conditioned Stimulus; EMDR = Eye Movement Desensitization and Reprocessing; EmoPics = Emotional Picture Set; FEBA = Facial Emotions for Brain Activation; IAPS = International Affective Picture System; M = Mean; N = Sample Size; NA = Non-Applicable; NFE = Novelty-Facilitated Extinction; SD = Standard Deviation; US = Unconditioned Stimulus.

a All participants are adults. Diseases included in this study are affective disorders (MDD, OCD, anxiety, PTSD, bipolar, etc.).

b SD was only available for the recruited sample but not the analyzed sample.

^*^Studies both implicit and explicit emotion regulation.

**Table 2:** Summary of Explicit Emotion Regulation Studies Meeting the Inclusion Criteria

**Authors**	**Task**	**Type of Emotion Regulation**	**Nhealthy (%Female)**	**Ndisease (%Female)**	**AgeHealthy** ^**a**^ **M (SD)**	**AgeDisease** ^**a**^ **M (SD)**	**Intervention**	**Stimuli**
Bastiaansen et al., 2018	Reappraisal	Controlled	69 (100%)		20.79 (1.84)		NA	Emotional images (IAPS)
Benzait et al., 2023	Reappraisal	Controlled	17 (35.3%)		31.4 (9.1)		NA	Emotional images (IAPS)
Berboth et al., 2021	Reappraisal	Controlled	25 (84%)		22.8 (3.3)		NA	Emotional images (IAPS)
Bezmaternykh et al., 2026	Reappraisal	Controlled	20 (100%)	20 (100%)	37.1 (8.4)	37.3 (11.1)	NA	Social images (COMPASS, EMOMadrid, GAPED, IAPS, NAPS, OASIS, SMID)
Bryant et al., 2021	Reappraisal	Controlled	24 (50%)	37 (45.9%)	35 (13.9)	41.4 (11.1)	NA	Emotional images (IAPS)
Butler et al., 2019	Reappraisal, Suppression	Controlled	27 (0%)	18 (0%)	32.7 (5.9)	28.3 (6.4)	NA	Emotional images (IAPS)
Che et al., 2015	Reappraisal	Controlled	29 (51.7%)		22.62 (1.59)		NA	Emotional images (CAPS)
Chrysikou et al., 2022	Reappraisal	Controlled	20 (70%)	20 (70%)	24 (4.38)	24.2 (6.31)	tDCS	Emotional images (IAPS)
Corbalan et al., 2015	Reappraisal	Controlled	17 (52.9%)	19 (52.6%)	41.4 (13.3)	41 (12.5)	NA	Emotional images (IAPS)
Davis et al., 2018	Reappraisal	Controlled	20 (100%)	16 (100%)	32.8 (11.8)	33.7 (10.6)	NA	Emotional images (IAPS)
De Wit et al., 2015	Reappraisal	Controlled	38 (52.6%)	43 (51.2%)	39.6 (11.4)	38.4 (10)	TMS	Emotional images (IAPS)
Denny et al., 2015	Reappraisal	Controlled	17 (70.6%)		24.1 (NA)		NA	Emotional images (IAPS)
Depue et al., 2016	Suppression	Controlled	21 (NA)		NA (NA)		NA	Emotional images (IAPS)
Diers et al., 2021	Reappraisal	Controlled	48 (66.7%)		24.6 (4.1)		NA	Emotional images (EmoPicS & IAPS)
Dorfel et al., 2014	Reappraisal, Suppression, Distraction	Controlled	74 (100%)		23.15 (NA)		NA	Emotional images (IAPS)
Ferri et al., 2016	Selective Attention	Controlled	42 (57.1%)		21.8 (3.62)		NA	Emotional images (IAPS)
Firk et al., 2018	Distraction	Controlled	26 (100%)		27 (5.3)		NA	Cry stimuli sound Distractor: Mental counting
*Fitzgerald et al., 2020	Reappraisal	Controlled	49 (67%)		25.24 (7.98)		NA	Emotional Images (IAPS)
Gaebler et al., 2014	Reappraisal	Controlled	23 (78.3%)	21 (76.2%)	30 (7.99)	30.5 (7.17)	NA	Emotional images (IAPS)
Gianaros et al., 2014	Reappraisal	Controlled	157 (51%)		42.7 (7.3)		NA	Emotional images (IAPS)
Goldin et al., 2019	Reappraisal	Controlled	35 (57.1%)		32.2 (8.9)		NA	Sentence triggering neutral or negative social situation
Graner et al., 2025	Reappraisal, Distraction	Controlled	62 (58.1%)		55.6 (1.5)		NA	Cue words of negative autobiographical memories from the past 15 years
Hallam et al., 2014	Reappraisal, Suppression	Controlled	20 (50%)		23 (NA)		NA	Sad or disgusting short videos (Gross, 1998 & YouTube)
Hallam et al., 2015	Reappraisal, Suppression	Controlled	40 (50%)		20 (1)		NA	Emotional images (IAPS)
He et al., 2023	Reappraisal	Controlled	117 (51.3%)		20.38 (NA)^b^		TMS	Asian young adults social inclusion or exclusion-related images (Zheng et al., 2022)
Hermann et al., 2017	Distraction	Controlled	27 (100%)		21.59 (2.58)		NA	Emotional images (IAPS)
Hermann et al., 2021	Reappraisal	Controlled	85 (100%)		23.15 (2.65)		NA	Emotional images (IAPS)
Hortelano et al., 2023	Reappraisal	Controlled	33 (51.5%)		21.85 (NA)		Lithium / Placebo	Emotional images (IAPS)
Huneke et al., 2026	Placebo, reappraisal	Controlled, automatic	40 (60%)		24.38 (4.88)		Naltrexone / Placebo	Emotional images (IAPS)
Jentsch et al., 2019	Reappraisal, Distraction	Controlled	64 (50%)		23.81 (3.28)		Cortisol / Placebo	Emotional images (IAPS)
Keller et al., 2022a	Reappraisal	Controlled	34 (50%)	55 (49.1%)	39.38 (12.1)	39.54 (NA) ^b^	NA	Emotional images (IAPS)
Kirk-Provencher et al., 2023	Reappraisal	Controlled	75 (56%)		20.9848 (NA)^b^		NA	Emotional images (IAPS)
Koch et al., 2019	Distraction	Controlled	40 (50%)	37 (43.2%)	40 (NA)^b^	40.2 (NA)^b^	Oxytocin / Placebo	Emotional images (IAPS)
Kohler et al., 2022	Reappraisal	Controlled	20 (55%)		40.4 (13.2)		NA	Emotional images (IAPS)
Kondo et al., 2023	Reappraisal	Controlled	17 (NA)	19 (NA)	41.4 (13.3)	41 (12.5)	NA	Emotional images (IAPS)
Korb et al., 2015	Reappraisal	Controlled	18 (55.6%)		27 (NA)		NA	Sound of neutral or angry Italian voices and shapes (Berlin et al., 2000)
Lee et al., 2021	Reappraisal	Controlled	27 (40.7%)		25.33 (2.75)		NA	Emotional images (IAPS)
Li et al., 2021	Distraction, Reappraisal, Suppression	Controlled	32 (50%)		19.63 (1.34)		NA	Emotional images (OASIS)
Lloyd et al., 2021	Reappraisal	Controlled	36 (55.6%)		73.2 (6.7)		NA	Emotional images (IAPS)
*Loeffler et al., 2019	Reappraisal	Controlled	26 (53.8%)	26 (53.8%)	35.31 (11.23)	35.27 (11.03)	NA	Faces (FACES)
Maier et al., 2020	Reappraisal	Controlled	38 (55.3%)		21.93 (NA)		NA	Emotional images (IAPS)
Morawetz et al., 2016a	Reappraisal	Controlled	59 (33.9%)		32.47 (11.25)		NA	Emotional images (IAPS) and emotional film clips
Morawetz et al., 2016b	Reappraisal	Controlled	23 (65.2%)		22.95 (3.57)		NA	Extreme sports and neutral films
Morawetz et al., 2016c	Reappraisal	Controlled	60 (50%)		30.48 (11.1)		NA	Emotional faces (FACES)
Morawetz et al., 2017a	Reappraisal	Controlled	23 (52.2%)		25.7 (5.95)		NA	Emotional images (IAPS)
Morawetz et al., 2017b	Reappraisal	Controlled	82 (39.02%)		30.77 (10.49)		NA	Emotional images (IAPS)
Morawetz et al., 2020b	Reappraisal	Controlled	35 (82.9%)		23.17 (3.44)		NA	Emotional images (IAPS)
Mulej Bratec et al., 2015	Reappraisal	Controlled	24 (100%)		24.8 (2.3)		NA	US: Negative images (IAPS) CS: Colored shapes
Murakami et al., 2015	Suppression	Controlled	21 (52.4%)		25.1 (5.5)		NA	Emotional images (IAPS)
Naor et al., 2020	Reappraisal	Controlled	33 (57.6%)		24.65 (1.76)		NA	Images of hands and feet in non-painful or painful scenarios
Navas et al., 2017	Reappraisal	Controlled	19 (NA)		NA (NA)^b^		NA	Emotional images (IAPS)
Nelson et al., 2015	Reappraisal	Controlled	22 (50%)		25.2 (5.8)		NA	Emotional faces (NimStim)
Otto et al., 2014	Reappraisal	Controlled	26 (100%)		24.88 (5.58)		NA	Non-Emotional or Emotional faces (NimStim)
Pan et al., 2023	Reappraisal	Controlled	105 (48.6%)		NA (NA)^b^		Cortisol / Placebo	Images (NAPS)
Paschke et al., 2016	Reappraisal	Controlled	108 (50.9%)		26.12 (3.7)		NA	Images (Emotional Picture Set)
Petersen et al., 2018	Reappraisal	Controlled	18 (100%)	18 (100%)	25.4 (6.99)	29.2 (7.24)	NA	Emotional images (IAPS)
Powers et al., 2020	Reappraisal	Controlled	34 (47.1%)		24.4 (3.6)		NA	Emotional images (IAPS)
Powers et al., 2022	Reappraisal	Controlled	40 (70%)		25.5 (4.7)		NA	Emotional images (IAPS)
Rabinak et al., 2014	Reappraisal	Controlled	21 (0%)	21 (0%)	34.81 (9.54)	30.24 (7.29)	NA	Emotional images (IAPS)
Radke et al., 2018	Reappraisal	Controlled	22 (40.9%)	22 (40.9%)	32.6 (10.9)	34.5 (9.9)	NA	Emotional faces (FACES)
Rehbein et al., 2021	Reappraisal	Controlled	29 (100%)		24.09 (0.58)		Valerate / Placebo	Emotional images (IAPS)
Reinecke et al., 2015	Reappraisal	Controlled	18 (77.8%)	18 (77.8%)	32.3 (12.1)	36.5 (13.8)	NA	Emotional images (IAPS)
Reinecke et al., 2018	Reappraisal	Controlled		28 (NA)		36 (NA)^b^	CBT immediate / Waiting	Emotional images (IAPS)
Rive et al., 2015	Reappraisal	Controlled	36 (69.4)	77 (63.6%).	40.2 (10.8)	42.56 (NA)^b^	NA	Emotional images (IAPS)
Robinson et al., 2026	Attention deployment	Controlled	28 (78.6%)		NA (NA)		NA	US: Electrical shocks CS: Neutral faces (NimStim), which were scrambled with place features during ER
Rubin-Falcone et al., 2020	Reappraisal	Controlled	19 (63%)	32 (63%)	32.9 (10)	34.8 (10.6)	CBT	Emotional images (IAPS)
Sarkheil et al., 2015	Reappraisal	Controlled	14 (57.1%)		NA (NA)		NA	Emotional images (IAPS)
Scharnowski et al., 2020	Reappraisal	Controlled	23 (47.8%)		27.7 (6.2)		NA	Emotional images (IAPS)
Shermohammed et al., 2017	Reappraisal	Controlled	54 (50%)		21.02 (NA)^b^		NA	Emotional images (IAPS)
Silvers et al., 2015b	Reappraisal	Controlled	13 (100%)		21.97 (NA)^b^		NA	Emotional images (IAPS)
Simsek et al., 2017	Reappraisal	Controlled	31 (100%)		22.22 (NA)^b^		NA	Emotional images (IAPS)
Sripada et al., 2014	Reappraisal	Controlled	49 (46.9%)		23.63 (1.3)		NA	Emotional images (IAPS)
Tamm et al., 2019	Reappraisal	Controlled	72 (NA)		NA (NA)		NA	Emotional images (IAPS)
Thorsen et al., 2019	Reappraisal	Controlled	57 (45.6%)	43 (51.2%)	38.47 (NA)^b^	37.58 (10.00)	NA	Emotional images (IAPS)
Tsujimoto et al., 2022	Reappraisal, Distraction	Controlled	50 (34%)		21.1 (1.9)		NA	Positive images (photoAC)
Uchida et al., 2015	Reappraisal	Controlled	62 (51.6%)		22.3 (1.6)		NA	Emotional images (IAPS)
van Dam et al., 2021	Reappraisal	Controlled	18 (66.7%)	19 (89.5%)	23.94 (4.57)	24.11 (5.53)	tDCS / Sham	Emotional images (IAPS)
Van der Meer et al., 2014	Reappraisal, Suppression	Controlled	40 (37.5%)		34.05 (NA)^b^		NA	Emotional images (IAPS)
Van der Meulen et al., 2017	Placebo	Automatic	30 (56.7%)		25.7 (5.5)		Analgesic cream & Control cream	Painful stimuli
van der Velde et al., 2015a	Reappraisal, Suppression	Controlled	51 (45.1%)		37.1 (10.3)		NA	Emotional images (IAPS)
van der Velde et al., 2015b	Reappraisal	Controlled	31 (48.4%)		22.6 (NA)^b^		NA	Emotional images (IAPS)
Wang et al., 2017	Reappraisal	Controlled	15 (53.3%)	12 (58.3%)	25.8 (5.89)	29.5 (8.46)	NA	Emotional images (IAPS)
Wang et al., 2022	Reappraisal	Controlled	19 (100%)		21.47 (2.91)		Experiential ER & Cognitive defusion training	Emotional images (IAPS)
Westbrook et al., 2023	Suppression, Distraction	Controlled	38 (NA)^b^		NA (NA)^b^		NA	Idiographic worry cues (The Worry and Rumination Interview)
Wu et al., 2021	Reappraisal	Controlled	27 (51.85%)		24.11 (2.33)		NA	Emotional images (IAPS)
Xin et al., 2020	Distancing	Controlled	65 (0%)		20.83 (2.6)		Oxytocin / Placebo	Emotional images (IAPS)
Zhang et al., 2018	Reappraisal	Controlled	17 (41.18%)	23 (56.52%)	32.47 (10.87)	40.91 (11.33)	NA	Emotional images (IAPS)

*Note.* This table contains all studies that met the inclusion criteria. Not all studies were further used in the analysis due to insufficient sample size for some contrasts (e.g., Healthy Control vs. Patient for ER). Abbreviations: CAPS = Native Chinese Affective Picture System; CBT = Cognitive Behavioral Therapy; COMPASS = The Complex Affective Scene Set; CS= Conditioned Stimulus; EMOMadrid = Emotional pictures database designed by Cerebro, Afecto y Cognición (CEACO); EmoPicS = Emotional Picture Set; ER = Emotional Regulation; FACES = FACES database by Ebner et al. (2010); GAPED = The Geneva Affective Picture Database; IAPS = International Affective Picture System; M = Mean; N = Sample Size; NA = Non-Applicable; NAPS = Nencki Affective Picture System; NimStim = The NimStem Set of Facial Expressions Dataset from macbrain.org/resources.htm; OASIS = Open Affective Standardized Image Set; photoAC = photo-ac.com; SD = Standard Deviation; SMID = The Socio-Moral Image Database; tDCS = Transcranial Direct Current Stimulation; TMS = Transcranial Magnetic Stimulation; US = Unconditioned Stimulus.

a All participants are adults. Diseases included in this study are affective disorders (MDD, OCD, anxiety, PTSD, bipolar, etc.).

b SD was only available for the recruited sample but not the analyzed sample.

^*^Studies both implicit and explicit emotion regulation.

The final sample of the ALE analysis consisted of 79 experiments (1101 foci) among 2961 healthy subjects for explicit ER and 29 experiments (255 foci) among 1329 subjects for implicit ER. The final inclusion for analysis did not contain coordinates of patient sample because the patient versus healthy control contrast did not have enough number of experiments to perform a robust ALE analysis as the explicit strategies only had 5 experiments (18 foci) and the implicit strategies only had 3 experiments (29 foci) respectively.

### ALE analysis among healthy subjects

#### The neural basis of explicit emotion regulation

In comparison to non-regulation conditions during which participants would naturally react to emotional stimuli, explicitly regulating these emotions typically yielded 14 clusters, as shown in Table 3. Most of the regions that consistently showed activations during explicit ER across different types of ER strategies were located in the frontal lobe (Fig. 2). The overall distribution of the clusters was widespread and bilateral, but predominantly left-lateralized in terms of total volume. The largest cluster (7512 mm^3^) was found in the left hemisphere. The most commonly reported regions included the inferior/superior/middle frontal gyri, as well as middle/superior temporal gyri, along with the insula, inferior parietal lobule, precuneus, medial frontal gyrus (MFG), sub-gyral regions, supramarginal gyrus, and cingulate gyrus.

**Table 3:** Convergence and Divergence Result Across Different Types of Emotion Regulation Strategies Among Healthy Subjects.

**Contrasts**	**Cluster Number**	**Cluster Size (mm** ^**3**^ **)**	**Peaks (BA)** ^**a**^	**Laterality**	**MNI coordinates**			***z*-score**
					**x**	**y**	**z**	
*Implicit Regulation > Non-regulation*								
	1	3280	Insula (NA)	R	34	24	−8	6.03
			Insula (13)	R	34	22	6	5.48
			Inferior frontal gyrus (13)	R	44	24	2	4.35
	2	3032	Insula (13)	L	−32	20	10	6.04
			Insula (NA)	L	−34	22	−4	5.45
	3	1448	Medial frontal gyrus (32)	R	4	14	46	4.84
	4	1280	Caudate (NA)	L	−8	4	6	5.13
			Lentiform nucleus (NA)	L	−12	6	−4	3.86
	5	1072	Cingulate gyrus (32)	R	6	24	30	4.50
			Cingulate gyrus (32)	L	−4	24	32	4.28
	6	968	Precuneus (7)	L	−26	−60	44	5.51
*Explicit Regulation > Non-regulation*								
	1	7512	Inferior frontal gyrus (47)	L	−46	28	−8	7.23
			Inferior frontal gyrus (47)	L	−48	34	−6	6.99
			Inferior frontal gyrus (45)	L	−52	22	12	6.92
			Insula (13)	L	−44	14	−2	3.38
	2	7448	Middle temporal gyrus (39)	L	−48	−60	28	6.31
			Inferior parietal lobule (40)	L	−48	−56	46	4.26
			Inferior parietal lobule (39)	L	−50	−62	44	3.89
			Precuneus (39)	L	−44	−70	40	3.25
	3	7184	Medial frontal gyrus (6)	L	−6	12	64	8.79
			Superior frontal gyrus (6)	R	16	20	58	5.39
	4	5656	Middle frontal gyrus (6)	L	−42	6	50	9.02
			Middle frontal gyrus (6)	L	−40	18	46	5.31
	5	4048	Middle temporal gyrus (NA)	L	−52	−38	−2	5.95
			Middle temporal gyrus (21)	L	−54	−30	−4	5.75
			Middle temporal gyrus (21)	L	−62	−40	−2	5.60
	6	3848	Inferior frontal gyrus (47)	R	48	22	−6	6.12
			Inferior frontal gyrus (45)	R	54	32	−6	4.84
			Sub-Gyral (10)	R	46	44	−6	3.31
	7	3768	Supramarginal gyrus (40)	R	56	−52	32	5.15
			Middle temporal gyrus (19)	R	50	−60	20	3.66
	8	1912	Middle frontal gyrus (8)	R	40	26	40	5.39
			Middle frontal gyrus (8)	R	36	36	34	3.12
	9	1384	Superior temporal gyrus (41)	R	48	−34	0	6.81
	10	1320	Cingulate gyrus (32)	L	−2	20	38	4.86
			Superior frontal gyrus (6)	L	−6	28	52	3.59
	11	1240	Superior frontal gyrus (8)	L	−12	50	38	4.74
			Superior frontal gyrus (9)	L	−16	50	34	4.69
	12	1216	Middle temporal gyrus (21)	L	−54	−2	−26	4.85
	13	1088	Superior temporal gyrus (38)	R	52	12	−24	4.86
			Middle temporal gyrus (21)	R	52	4	−24	4.31
	14	1080	Middle frontal gyrus (9)	R	36	48	22	4.42
			Superior frontal gyrus (9)	R	28	54	28	4.34
			Superior frontal gyrus (9)	R	18	56	28	4.06
*Explicit Regulation > Implicit Regulation*								
	1	6704	Superior temporal gyrus (39)	L	−48	−59.9	31.3	3.89
	2	5128	Middle frontal gyrus (6)	L	−41	13.1	50.1	3.89
			Precentral gyrus (6)	L	−47	2	56	3.72
	3	4736	Medial frontal gyrus (6)	L	−12	9.9	67.4	3.89
			Superior frontal gyrus (6)	L	−5	5.6	64.7	3.72
	4	3720	Middle temporal gyrus (NA)	L	−57	−36.6	−4.2	3.89
			Superior temporal gyrus (21)	L	−52	−26	−6	3.72
	5	2424	Inferior frontal gyrus (47)	L	−44	29.4	−13.6	3.72
			Inferior frontal gyrus (47)	L	−48	22	−14	3.19
	6	2424	Middle temporal gyrus (39)	R	47	−60.3	29.8	3.89
			Middle temporal gyrus (39)	R	47	−62.7	20.7	3.72
			Middle temporal gyrus (39)	R	55	−64	25.3	3.43
	7	1608	Inferior frontal gyrus (44)	L	−56	20	8	2.88
			Inferior frontal gyrus (45)	L	−54	24	10	2.83
			Inferior frontal gyrus (44)	L	−50	18	10	2.76
			Inferior frontal gyrus (44)	L	−58	22	14	2.61
	8	1184	Superior temporal gyrus (38)	L	−51	9.3	−21.3	3.89
			Middle temporal gyrus (21)	L	−52	6	−32	2.85
	9	1008	Inferior frontal gyrus (47)	R	46	35	−12	2.95
	10	992	Superior temporal gyrus (38)	R	50	2	−26	2.81
			Superior temporal gyrus (38)	R	50	0	−22	2.76
			Middle temporal gyrus (21)	R	53	3.6	−23.2	2.63
			Sub-Gyral (38)	R	52	6	−22	2.64
			Superior temporal gyrus (38)	R	49	13.3	−26	2.38
	11	952	Superior frontal gyrus (8)	L	−6	52	38	2.51
			Superior frontal gyrus (8)	L	−10	53	38	2.51
			Superior frontal gyrus (8)	L	−14	52	32	2.47
			Superior frontal gyrus (8)	L	−14	52	40	2.34
	12	840	Middle frontal gyrus (9)	R	42	30	30	2.88
			Middle frontal gyrus (8)	R	39	31	37	2.24
	13	528	Superior temporal gyrus (41)	R	46	−30	4	2.13
			Superior temporal gyrus (41)	R	44	−34.6	3.7	2.13
	14	416	Superior frontal gyrus (6)	R	20	26	58	2.55
			Superior frontal gyrus (6)	R	14	24	54	2.12
			Superior frontal gyrus (6)	R	20	18	62	1.95
*Implicit Regulation > Explicit Regulation*								
	1	2280	Claustrum (NA)	L	−28	18	10	3.29
			Claustrum (NA)	L	−28	22	12	3.19
			Claustrum (NA)	L	−26	24	−4	2.56
			Insula (13)	L	−34	20	0	2.55
	2	1888	Claustrum (NA)	R	30	24	−6	3.09
			Claustrum (NA)	R	30	26	4	2.52
			Insula (13)	R	38	24	2	2.51
			Insula (13)	R	36	26	6	2.49
	3	992	Cingulate gyrus (24)	R	12	12	44	2.85
			Medial frontal gyrus (32)	M	0	14	48	2.27
			Cingulate gyrus (24)	L	−4	8	46	2.19
			Cingulate gyrus (24)	L	−8	8	46	2.13
	4	928	Caudate (NA)	L	−6	6	8	3.09
			Lentiform nucleus (NA)	L	−12	4	−2	2.05
	5	920	Precuneus (7)	L	−22	−62	40	2.54
			Precuneus (7)	L	−18	−64	42	2.47
			Superior parietal lobule (7)	L	−28	−56	48	2.43
			NA (NA)	L	−26	−53	43	2.03
	6	744	Cingulate gyrus (32)	R	12	26	26	3.54
			Cingulate gyrus (24)	R	4	20	27	3.24
								
*Conjunction of Explicit and Implicit Regulation*								
	1	448	Insula (13)	L	−36	22	−4	NA
	2	320	Inferior frontal gyrus (13)	R	46	24	2	NA
	3	112	Cingulate Gyrus (32)	L	−4	24	32	NA
	4	48	Insula (47)	R	36	20	−10	NA
	5	16	Insula (13)	R	42	22	−4	NA
	6	16	Medial frontal gyrus (32)	R	2	16	44	NA
	7	8	Medial frontal gyrus (6)	R	2	18	46	NA

*Note.* Abbreviations: L = Left; M = Middle of the Brain; MNI = Montreal Neurological Institute; NA = Non-Applicable; R = Right.

a Peaks are reported in Broadmann area, if available. Areas that no grey matter are found are reported in NA. Numbers in the bracket represents the number in the Broadmann atlas. Regions where the number in the Broadmann atlas was not reported in the ALE output were denoted as NA.

**Table 4:** Robustness of Significant Clusters of Contrast and Conjunction Analysis Conducted with Leave-One-Out Analsysis

**Contrasts**	**Cluster Number**	***N*** _**voxel**_	**Peaks (BA)** ^**a**^	**Laterality**	**Overlap Rate**	**DSC**	**Survival Rate**	**Most Disruptive Study**
*Explicit Regulation > Implicit Regulation*								
	1	838	Superior temporal gyrus (39)	L	100%	0.93	100%	Wu et al., 2021
	2	641	Middle frontal gyrus (6)	L	100%	0.93	100%	Wu et al., 2021
			Precentral gyrus (6)	L				
	3	592	Medial frontal gyrus (6)	L	100%	0.95	100%	Wu et al., 2021
			Superior frontal gyrus (6)	L				
	4	465	Middle temporal gyrus (NA)	L	100%	0.94	100%	Huneke et al., 2026
			Superior temporal gyrus (21)	L				
	5	303	Inferior frontal gyrus (47)	L	100%	0.94	98.1%	Morawetz et al., 2016a
			Inferior frontal gyrus (47)	L				
	6	303	Middle temporal gyrus (39)	R	100%	0.88	100%	Hallam et al., 2015
			Middle temporal gyrus (39)	R				
			Middle temporal gyrus (39)	R				
	7	201	Inferior frontal gyrus (44)	L	100%	0.90	99.1%	Bezmaternykh et al., 2026
			Inferior frontal gyrus (45)	L				
			Inferior frontal gyrus (44)	L				
			Inferior frontal gyrus (44)	L				
	8	148	Superior temporal gyrus (38)	L	100%	0.85	100%	Berboth et al., 2021
			Middle temporal gyrus (21)	L				
	9	126	Inferior frontal gyrus (47)	R	100%	0.85	100%	Berboth et al., 2021
	10	124	Superior temporal gyrus (38)	R	100%	0.91	100%	Gianaros et al., 2014
			Superior temporal gyrus (38)	R				
			Middle temporal gyrus (21)	R				
			Sub-Gyral (38)	R				
			Superior temporal gyrus (38)	R				
	11	119	Superior frontal gyrus (8)	L	100%	0.81	100%	Tamm et al., 2019
			Superior frontal gyrus (8)	L				
			Superior frontal gyrus (8)	L				
			Superior frontal gyrus (8)	L				
	12	105	Middle frontal gyrus (9)	R	99.1%	0.80	99.1%	Tamm et al., 2019
			Middle frontal gyrus (8)	R				
	13	66	Superior temporal gyrus (41)	R	100%	0.85	100%	Morawetz et al., 2017b
			Superior temporal gyrus (41)	R				
	14	52	Superior frontal gyrus (6)	R	98.1%	0.82	98.1%	Morawetz et al., 2020b
			Superior frontal gyrus (6)	R				
			Superior frontal gyrus (6)	R				
*Implicit Regulation > Explicit Regulation*								
	1	285	Claustrum (NA)	L	100%	0.95	100%	Stickel et al., 2019
			Claustrum (NA)	L				
			Claustrum (NA)	L				
			Insula (13)	L				
	2	236	Claustrum (NA)	R	100%	0.93	100%	Wroblewski et al., 2022
			Claustrum (NA)	R				
			Insula (13)	R				
			Insula (13)	R				
	3	124	Cingulate gyrus (24)	R	100%	0.83	97.2%	Hennings et al., 2020
			Medial frontal gyrus (32)	M				
			Cingulate gyrus (24)	L				
			Cingulate gyrus (24)	L				
	4	116	Caudate (NA)	L	100%	0.82	100%	Wroblewski et al., 2022
			Lentiform nucleus (NA)	L				
	5	115	Precuneus (7)	L	100%	0.95	100%	Chechko et al., 2014
			Precuneus (7)	L				
			Superior parietal lobule (7)	L				
			NA (NA)	L				
	6	93	Cingulate gyrus (32)	R	99.1%	0.96	99.1%	Dunsmoor et al., 2019
			Cingulate gyrus (24)	R				
*Conjunction of Explicit and Implicit Regulation*								
	1	56	Insula (13)	L	100%	0.85	100%	Huneke et al., 2026
	2	40	Inferior frontal gyrus (13)	R	97.2%	0.71	97.2%	Miedl et al., 2020
	3	14	Cingulate Gyrus (32)	L	0%	0.00	0%	Dorfel et al., 2014
	4	6	Insula (47)	R	0%	0.00	0%	Dorfel et al., 2014
	5	2	Insula (13)	R	94.4%	0.06	94.4%	Goldin et al., 2019
	6	2	Medial frontal gyrus (32)	R	0%	0.00	0%	Dorfel et al., 2014
	7	1	Medial frontal gyrus (6)	R	0%	0.00	0%	Dorfel et al., 2014

*Note.* Abbreviations: DSC = Dice Similarity Coefficient; L = Left; M = Middle of the brain; R = Right.

a Peaks are reported in Broadmann area, if available. Areas that no grey matter are found are reported in NA. Numbers in the bracket represents the number in the Broadmann atlas. Regions where the number in the Broadmann atlas was not reported in the ALE output were denoted as NA.

**Figure 2:**
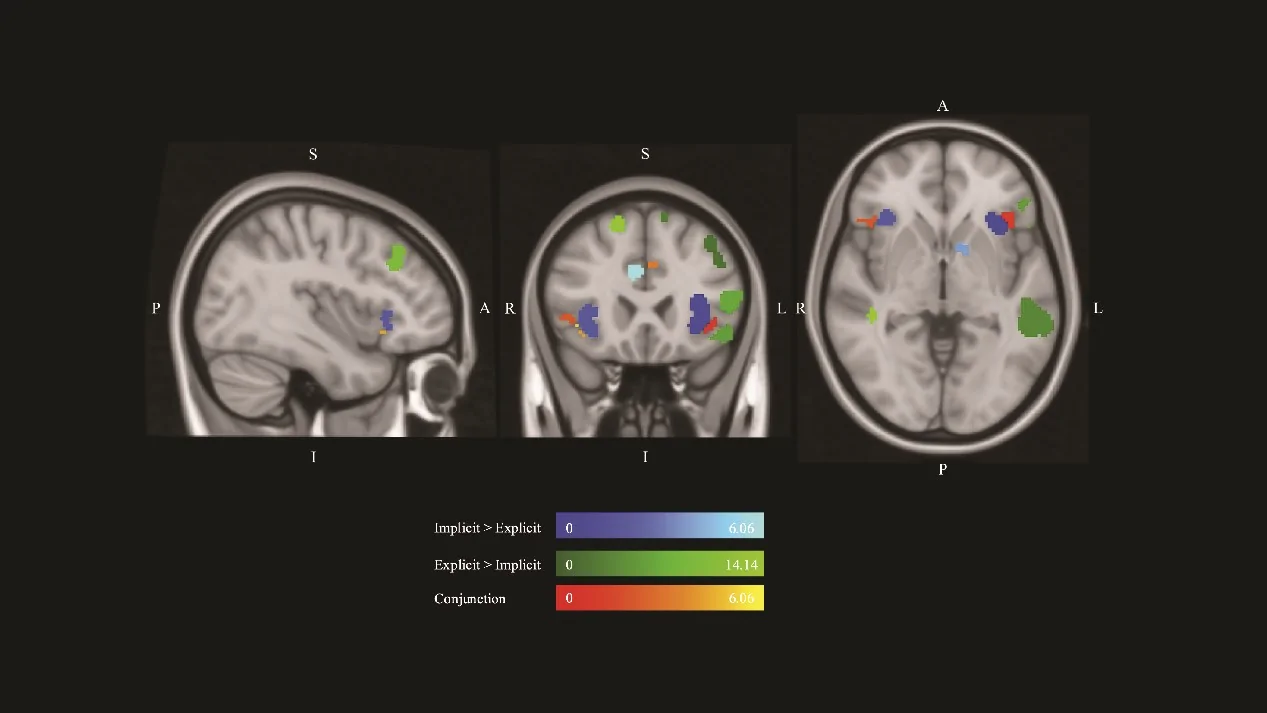
Regions independently and jointly responsible for explicit and implicit emotion regulation. *Note*. Regions in red represent regions that are responsible for both explicit and implicit emotion regulation. Regions in green denote for regions responsible for explicit emotion regulation whereas regions in blue denote for regions responsible for implicit emotion regulation.

#### The neural basis of implicit emotion regulation

Compared to non-emotion-regulation conditions, implicit ER conditions evoked six clusters (Table 3). The insula and cingulate gyrus were frequently activated when emotions were implicitly regulated. Additionally, specific clusters were identified in the inferior frontal gyrus, MFG, caudate, lentiform nucleus, and precuneus. The overall distribution of the clusters was bilateral. The largest cluster (3280 mm^3^) was found in the right hemisphere.

#### Different neural basis of explicit and implicit emotion regulation

As shown in Table 3, we found 14 clusters that were more commonly engaged in explicit ER strategies than in implicit ER strategies. Most clusters were found in the frontal and temporal lobes. Overall, for the contrast *Explicit ER > Implicit ER*, the distribution of the significant clusters was bilateral, though the left hemisphere showed a more pronounced dominance in total activation volume. The largest cluster (6704 mm^3^) was found in the left hemisphere, along with the next several largest clusters (clusters 2–5). The middle temporal gyrus, superior frontal gyrus and superior temporal gyrus were commonly reported regions that presented bilaterally. Meanwhile, activation in the inferior frontal gyrus demonstrated prominent left-sided lateralization.

Regarding the contrast *Implicit ER > Explicit ER*, six clusters showed unique contributions to implicit ER, rather than explicit ER. Compared to explicit ER strategies, implicit ER strategies were more reliant on regions in the subcortical and limbic areas such as the claustrum, insula, caudate, lentiform nucleus, and cingulate gyrus, though regions in the frontal and parietal lobes, specifically the MFG, precuneus, and superior parietal lobule, were also reported. In contrast to the *Explicit ER > Implicit ER* contrast, which showed strong left-hemisphere dominance in terms of total volume, implicit regulation yielded a much more balanced, bilateral cluster distribution when directly contrasted with explicit strategies. Despite this general symmetry, the maximum cluster volume (2280 mm^3^) mapped to the left hemisphere. Key areas driving this effect included the claustrum and insula, both of which exhibited distinct bilateral engagement.

#### Shared neural basis of explicit and implicit emotion regulation

Conjunction analysis revealed seven clusters that consistently contributed to both explicit and implicit ER. The overall distribution of the clusters commonly activated by both types of ER strategies was primarily bilateral. Consequently, the left hemisphere contained the largest overlapping cluster (448 mm^3^), closely followed by a smaller cluster in the right hemisphere (320 mm^3^). Within this conjunction, the insula and the inferior frontal gyrus emerged as distinct bilateral hubs, while additional shared activation was observed bilaterally in the medial wall, encompassing the left cingulate gyrus and right MFG.

#### Generally robust clusters according to the LOO analysis

Overall, results from the LOO analysis indicated high robustness and spatial stability across the majority of neural regions identified with GingerALE. Regarding the contrast analyses, identified regions demonstrated high resilience against dataset variations. For the *Explicit ER > Implicit ER* contrast, all 14 identified clusters demonstrated robust neural recruitment patterns with survival and overlap rates exceeding 98%. Specifically, 10 out of the 14 clusters achieved a 100% strict survival rate by meeting the following simultaneous criteria: minimum cluster size, ≥10% spatial coverage, and ≤ 15 mm peak drift across all LOO iterations. These clusters also demonstrated high spatial reproducibility across iterations, with DSCs ranging from 0.80 to 0.95.

Similarly, the regions identified in the *Implicit ER > Explicit ER* contrast demonstrated high stability across the LOO iterations. All clusters show overlap rate higher than 99%. Of the six clusters, four maintained a 100% strict survival rate across all iterations. The remaining two clusters showed survival rates higher than 97%. These clusters also maintained strong spatial alignment, with DSC values ranging from 0.82 to 0.96.

Regarding the conjunction analysis, three out of the seven clusters showed overlap and strict survival rates higher than 94%. The two larger robust clusters (≥40 voxels) further demonstrated robust spatial reproducibility with DSCs greater than 0.70 (Cluster 1: DSC = 0.85; Cluster 2: DSC = 0.71). In contrast, the third surviving cluster (Cluster 5), despite its high survival rate of 94.4%, consisted of only 2 voxels and exhibited poor spatial reproducibility (DSC = 0.06). The remaining four small clusters (all comprising 14 voxels or fewer) showed 0% survival and overlap rates, indicating that these micro-clusters were driven by idiosyncratic study effects and possessed poor spatial reproducibility.

## Discussion

In this meta-analysis, we used ALE to study the convergence and divergence of explicit and implicit ER. We hypothesized explicit ER to recruit more regions responsible for goal-directed behaviors, cognitive control, and consciousness/awareness in comparison to implicit ER. We further hypothesized implicit ER to evoke more regions responsible for emotional responses and fewer regions responsible for cognitive control, compared to explicit ER. Finally, we hypothesized both types of ER strategies to have overlaps in regions responsible for emotional processing and generation.

Our findings are in line with our hypotheses which showed more activations in the frontal and temporal lobe for explicit ER compared to implicit ER and more activations in the subcortical areas for implicit ER compared to explicit ER. These results build upon foundational explicit ER meta-analyses by confirming the core frontoparietal network’s role. Interestingly, our findings, which were derived from a more refined topographical mapping that directly contrasts these established networks with implicit processes, align well with recent meta-analytic work of implicit ER – using different definitions than ours – conducted among clinical populations (Dalton et al. 2025). This article highlights the critical role of subcortical–cortical integration in implicit regulation. Furthermore, our findings for explicit ER also align robustly with established neuroimaging consensus on top-down control. Specifically, our *Explicit ER > Implicit ER* contrast revealed extensive, heavily left-lateralized clusters across the middle and superior frontal gyri (extending into the dlPFC), the inferior frontal gyrus, and the middle temporal gyrus. Comprehensive meta-analyses dissecting explicit ER strategies have consistently reaffirmed this distinct prefrontal lateralization, emphasizing the central role of left-sided prefrontal and temporal regions in deploying top-down cognitive control (Morawetz et al. 2017, White et al. 2023). Because explicit ER heavily relies on antecedent-focused strategies like cognitive reappraisal, this robust left-lateralized network likely reflects the demanding semantic re-representation and internal verbal processing required to actively alter the meaning of an affective stimulus (Buhle et al. 2014).

### Explicit emotion regulation is goal-directed

Our ALE analysis yielded many regions in the frontal and temporal lobes for the contrast *Explicit ER > Implicit ER*. These activations were exclusively cortical, with a denser distribution in the lateral (e.g. inferior and middle frontal/temporal gyri) rather than the medial parts of the hemispheres. This result aligns with findings from prior literature suggesting contributions from the left inferior frontal gyrus, middle temporal gyrus, and inferior parietal lobule to successful ER via goal setting (Morawetz et al. 2016).

Regions found in our analysis are related not only to ER goals but also to general goal-directed behaviors (Dixon et al. 2017). Notably, these lateral regions overlap with areas previously identified via decoding methods as the neural bases of ‘reappraisal-only’ processes (regions uniquely driving regulation, such as the middle temporal gyrus and inferior parietal lobule) and ‘common appraisal’ (regions bridging regulation and emotion generation, such as the dorsolateral prefrontal cortex; Bo et al. 2024). Though Bo et al. (2024) found more extensive cortical activations than we did, they focused solely on one explicit ER strategy, whereas we synthesized multiple implicit and explicit paradigms. The overlaps between our findings and the consistencies in topographical distribution – specifically the reliance on lateral rather than medial cortical regions – underscore the robustness of our results. It demonstrates that the deliberate goal-directed regulation of emotions consistently evokes lateral cortical control networks, providing strong neural support for Braunstein et al. (2017)‘s classification.

### Implicit emotion regulation targets the emotional aspects of regulation but not the awareness of goals

Our ALE results showed activations in subcortical areas for the contrast *Implicit ER > Explicit ER*, alongside distinct cortical engagement. The peaks reported in our ALE analysis align with prior literature on emotion processing; for instance, the insula is widely regarded as the interface between perception, emotion, and cognition (R. Zhang et al. 2024). Crucially, the specific cortical and subcortical regions identified in our *Implicit ER > Explicit* ER contrast – namely the MFG, cingulate gyrus, superior parietal lobule, and lentiform nucleus – strongly overlap with the ‘non-modifiable’ emotion generation network identified by Bo et al. (2024). Using decoding methods, Bo et al. found that these specific regions (e.g. the medial prefrontal cortex, midcingulate, superior parietal cortices, and putamen) are fundamentally responsible for generating emotional responses but remain entirely unaffected by explicit regulation strategies like reappraisal. Therefore, this topographical overlap suggests that implicit ER uniquely targets the core affective and reactivity-based aspects of emotion generation that effortful, explicit regulation fails to downregulate.

Interestingly, the claustrum reported by the ALE result has also been suggested to act as a hub for integrating multisensory information for the production of a unitary conscious representation (Calarco 2024, Liaw and Augustine 2023, Yin et al. 2016). Similarly, caudate, a region essential for consciousness (Rohaut et al. 2019), was consistently reported when contrasting non-regulation or explicit ER from implicit ER.

This result again supports Braunstein et al. (2017)'s classification of ER according to the explicitness (consciousness) of ER goal and degree of ER effort. The difference between implicit and explicit ER may indeed lie in the level of consciousness involved. Therefore, the transferrable effect of one type of ER training on another may depend on the level of integration between multisensory information which contributes to the unitary conscious representation. Training on such an integration ability may be a future therapeutic point.

In addition to subcortical involvement, our analysis identified specific cortical regions engaged during implicit ER, most notably the MFG. This region is frequently implicated in the automatic exertion of cognitive control during affective interference (Etkin et al. 2011). Notably, recent meta-analytic evidence incorporating pre-2014 literature has also demonstrated significant alterations in MFG activation during implicit ER across clinical populations, despite utilizing different task paradigms and definitions than our study (Dalton et al. 2025). Because the MFG supports implicit ER in both healthy individuals (as shown in our results) and clinical populations, it represents a crucial, transdiagnostic node across various task modalities. Consequently, the automatic regulation of affective interference likely serves as a fundamental construct underlying implicit ER.

### Only a highly specific network robustly shows activations for both implicit and explicit emotion regulation

The conjunction analysis initially yielded seven clusters; however, our rigorous LOO sensitivity analysis revealed a small, highly specific overlap between these two types of strategies. Specifically, only the bilateral insula and right inferior frontal gyrus demonstrated high spatial robustness across dataset variations, while medial structures identified in the main analysis (such as the cingulate and medial frontal gyri) failed to survive. Nevertheless, the robust regions reported by our ALE analysis include a core interface between sensation, emotion, and cognition (i.e. the insula), and a key region for explicit ER and cognitive control (i.e. the right inferior frontal gyrus). This suggests that coordination specifically between the insula and the inferior frontal gyrus is essential for ER in general, regardless of the explicitness of the regulation goal. The final output (implicit or explicit ER) may thereby depend on the degree of influence from the explicit ER areas on the integration of sensation, emotion, and cognition within the insula. The widely reported interaction between prefrontal control regions and insular reactivity may be the fundamental mechanism underlying ER processes in general (Ochsner and Gross 2008).

### Explicit and implicit emotion regulation show distinct hemispheric lateralization

Interestingly, for the *Explicit ER > Implicit ER* contrast, the largest activated cluster was found in the left hemisphere, specifically in the left middle/superior frontal gyrus, left inferior frontal gyrus and left middle temporal gyrus. Previous literature has demonstrated a content-specific lateralization in the prefrontal cortex during ER, with the left DLPFC being preferentially engaged in verbal and goal-directed regulation tasks (White et al. 2023). Our meta-analytic findings suggest that this left-lateralized preference for explicit ER extends beyond the DLPFC into broader regions involved in language and semantic processing (e.g. left inferior frontal gyrus, left middle temporal gyrus; Bartha et al. 2003; J. Zhang et al. 2025b), as well as executive functions (e.g. left middle/superior frontal gyrus; Boisgueheneuc et al. 2006; Xu et al. 2024). This left-lateralized dominance for large clusters in explicit ER, in contrast to implicit ER, may reflect the reliance on verbal and semantic processes during explicit ER strategies such as cognitive reappraisal, which involves internal processing of the semantic content of the emotional stimuli (Buhle et al. 2014).

In contrast, the *Implicit ER > Explicit ER* contrast showed a more balanced distribution of clusters across the two hemispheres, involving key regions such as the claustrum and the cingulate gyrus. Recently, bilateral activation in the claustrum that extended to the insula was found during implicit ER tasks, such as priming, among patients with mood disorders (Dalton et al. 2025). Though our final analysis focused on a healthy population instead of patients with mood and/or anxiety disorders due to the limited number of studies in the latter population, the consistent involvement of the bilateral claustrum in implicit ER across healthy and clinical populations highlights its essential role in implicit ER. Furthermore, given that processing emotional stimuli is essential during implicit ER strategies/tasks, and the fact that most implicit strategies/tasks use negatively valenced stimuli, the bilateral engagement of the cingulate gyrus may be related to the processing of negative emotional stimuli during implicit ER (Palomero-Gallagher and Amunts 2022).

The conjunction analysis, following our robustness checks, revealed a highly localized revealed a balanced, bilateral cluster distribution rather than strict lateralization. The insula, a key region for the integration of sensation, emotion, and cognition, showed bilateral activation in the conjunction analysis. Interestingly, bilateral insula activation has been found in response to all emotional stimuli, particularly towards negative emotional stimuli, as well as during the experience of emotions (Duerden et al. 2013). Since explicit and implicit ER strategies/tasks often involve regulating emotions that are evoked by negative emotional stimuli (e.g. screams, negative images, electric shocks) – either on demand (explicit ER) or implicitly for better task performance (implicit ER) – the bilateral activation of the insula is thus not surprising. In contrast to the bilateral insula, the inferior frontal gyrus showed primarily right-sided laterality in the conjunction analysis. The right inferior frontal gyrus is preferentially involved in the processing of affective content and negative affect (White et al. 2023), both of which are commonly involved in the stimuli used across explicit and implicit ER paradigms.

### Influence of contrast design on resulting activation patterns

One potential reason why distinct regions responsible for explicit rather than implicit ER were found primarily in cortical areas of the frontal and temporal lobes, whereas implicit ER uniquely engaged mostly subcortical regions, stems from differences in the fMRI contrasts used to capture the neural basis of these two types of ER. Specifically, contrasts used for explicit ER typically involve comparing an active regulation condition against the non-regulation of an emotional stimulus (e.g. *regulate negative > watch negative*). Since emotional stimuli are presented in both conditions of the contrast, this subtraction effectively partials out regions responsible for basic emotional processing, isolating cortical regions, which are typically involved in goal-directed behaviors and ER goals, for explicit ER. Therefore, emotion processing regions are still likely to be actively involved during explicit ER. Future studies can consider contrasting explicit ER conditions against a non-emotional baseline (e.g. *regulate negative > regulate neutral*) to better capture the emotional aspects of explicit ER and to further clarify the differences in the neural basis of explicit and implicit ER.

Conversely, implicit ER paradigms frequently involve shifting attention away from emotional distractors or executing automatic responses, often contrasting an emotional condition against a non-emotional baseline (e.g. *emotional no-go > neutral no-go*, or *incongruent > congruent*). As a result, the neural maps for implicit ER likely capture differences in the differentially valenced conditions, reflecting processes such as attentional reorienting during the confrontation with differently valenced stimuli (supported by the precuneus and superior parietal lobule; Cavanna and Trimble 2006; Q. Zhang et al. 2025a). Given the differences in stimulus valence, the involvement of regions heavily involved in affective processing, including the basal ganglia, MFG, cingulate gyrus, and claustrum, is unsurprising.

### Clinical implications for psychopathology and interventions

Although we were unable to perform ALE analysis among our target clinical population nor contrasting between clinical and healthy population, the identified divergence in the neural bases of explicit and implicit ER has some implications for clinical practice. Being a transdiagnostic factor in psychopathology, dysfunctions in ER are prevalent in psychiatric disorders, including mood and anxiety disorders (Aldao et al. 2010, Sloan et al. 2017). Patients show deficits in both effortful cognitive control over emotions and automatic emotional reactivity (Gyurak et al. 2011). Including ER as one of its core elements, common psychotherapies such as CBT mostly focus on the explicit ER end by training patients to consciously identify, evaluate, and cognitively reframe maladaptive thoughts (e.g. utilizing cognitive reappraisal). In our meta-analysis, we showed that explicit ER heavily recruits left-lateralized frontotemporal and parietal networks associated with semantic processing and goal-directed behavior. This suggests that the success of CBT may depend heavily on the structural and functional integrity of these cortical networks. Targeting these functional networks with precision-guided non-invasive brain stimulation, along with psychotherapy, might augment the effect of psychotherapy alone. In fact, some preliminary evidence already showed that combining transcranial magnetic stimulation or transcranial direct current stimulation over the prefrontal cortex with concurrent cognitive-behavioral interventions yields greater improvements in ER and symptom reduction than psychotherapy alone (Donse et al. 2018).

Besides issues with explicit ER, the clinical population also exhibits profound dysregulation that is automatic and independent of conscious regulatory goals (Joormann and Gotlib 2010, Tracy et al. 2014), driven by hyper-reactivity in subcortical and limbic networks according to our meta-analysis. Our finding that implicit ER uniquely recruits bilateral subcortical integration hubs – most notably the claustrum, insula, and cingulate gyrus – suggests that explicitly focused interventions may not sufficiently target the neural mechanisms driving automatic emotional responses. Consequently, interventions designed to enhance implicit ER, such as cognitive bias modification or implicit extinction learning, may be necessary to directly modulate these subcortical circuits. Furthermore, novel therapeutic approaches like real-time fMRI neurofeedback or non-invasive brain stimulation could potentially target the insular-cortical network identified in our conjunction analysis, fostering better coordination between top-down regulatory goals and bottom-up sensory-emotional integration. Ultimately, assessing a patient’s specific deficits across both explicit (cortical) and implicit (largely subcortical) ER domains may help tailor more personalized, neurobiologically informed treatment plans.

## Limitations and future directions

Although this meta-analysis followed strict inclusion and exclusion criteria, as well as a stringent threshold for performing the ALE analysis, there are some limitations that may restrict the generalizability of the results. First, even though we included articles studying ER among patients with mood and/or anxiety disorders, we did not have a sufficient sample size (only 12 studies) to perform an ALE specifically among patient populations. Therefore, our results are currently limited to healthy populations.

Second, we noticed a disproportional distribution in the number of different explicit ER strategies. Specifically, the number of reappraisal studies significantly outnumbered (by at least seven times) those of other explicit ER strategies, such as suppression, distraction, and selective attention. Therefore, it is possible that the main effect of *Explicit Regulation > Non-regulation* and the consequent contrast analysis between implicit and explicit ER is driven mainly by the effect of reappraisal. Future research should investigate a broader range of ER strategies other than cognitive reappraisal to ensure these findings generalize across different regulatory approaches.

Third, our meta-analysis combined studies utilizing both positive and negative emotional stimuli. Distinct valences can elicit divergent neural responses; for instance, positive and negative emotions leverage separate functional networks, such as distinct recruitment of the cuneus and cingulate gyrus versus the parahippocampal and fusiform gyri (Feng et al. 2024). By collapsing across valences to maintain statistical power, we may have masked valence-specific neural networks underlying ER. Future meta-analyses should consider valence-specific subgroup analyses when a sufficient volume of literature becomes available.

Fourth, while the primary meta-analysis used GingerALE, the LOO sensitivity analysis utilized NiMARE for computational efficiency. Consequently, minor spatial variations at cluster peripheries during LOO iterations may partially reflect subtle algorithmic differences between the software environments (e.g. differing brain masking volumes affecting FWE thresholds) rather than true instability from dataset exclusion.

Lastly, to maintain methodological rigor, we applied strict inclusion and exclusion criteria for study selection following a stringent definition of explicitness – the presence of ER goals (Braunstein et al. 2017). Consequently, certain implicit ER paradigms that were included in other meta-analyses (e.g. Dalton et al. 2025) – such as the bar orientation task – were omitted. While this stringent approach minimized confounding variables, it is a limitation as we may have missed potentially relevant studies, restricting the generalizability of our findings across the full spectrum of implicit regulatory processes.

Based on our ALE findings, future research should explore the potential of improving ER by targeting the shared neural basis of explicit and implicit ER. Despite the limited spatial overlap between explicit and implicit ER, the few overlapping regions (e.g. the insula and inferior frontal gyrus) are key hubs responsible for affect processing and the integration of sensation, emotion, and cognition, which are essential for both types of ER. Future studies could utilize real-time fMRI neurofeedback or non-invasive brain stimulation to target these shared regions in order to augment the effects of ER training (Cohen and Ochsner 2018) and to test whether such augmentation in one type of ER could also enhance the other.

## Conclusion

In conclusion, we found more involvement of the prefrontal regions for explicit ER compared to implicit ER. Implicit ER seems to recruit subcortical and cortical regions responsible for emotion processing and consciousness generation. Both implicit and explicit ER depends on regions responsible for the integration of sensation, emotion, and cognition, as well as regions conscious emotion control.

## Supplementary Material

supplementary_file_for_export_3_kvag013

## Data & code availability

All coordinates extracted from eligible articles for ALE analysis, as well as the code used for LOO sensitivity analysis, are publicly available and can be assessed from https://osf.io/zm792/files. This meta-analysis did not develop any code for ALE analysis. GingerALE was used and results can be validated following the parameter settings listed under the Method section of this manuscript, using the complied txt files as inputs.
