# Supplementary material for: The neural basis of explicit and implicit emotion regulation: a coordinate-based meta-analysis
Source: Oxf Open Neurosci. 2026 Sep 18;5:kvag013. doi: 10.1093/oons/kvag013 (PMC13630458; doi:10.1093/oons/kvag013)
Supplement: Supplementary file 1 [file supplementary_file_for_export_3_kvag013.docx]

This paper was peer reviewed at another journal before being transferred, and permission was not received to publish those reviews.

| **OXFNSC-2026 014** | **26-May-2026**   Dear Dr. Lam,  Thank you for submitting your manuscript OXFNSC-2026-014 entitled "The Neural Basis of Explicit and Implicit Emotion Regulation: A Coordinate-Based Meta-Analysis" to Oxford Open Neuroscience. Your manuscript has been seen by two reviewers with relevant expertise. The comments of the reviewer(s) are included at the bottom of this letter.  Both reviewers have provided helpful comments and find the manuscript potentially of interest. Therefore, I invite you to respond to the reviewer(s)' comments and revise your manuscript within the next 60 days from today date.  Both reviewers have made suggestions regarding detail and clarity, particularly in the Discussion, which you should carefully consider. The reviewers have also made some suggestions for further analyses. Reviewer 1 suggests including a sensitivity analysis and reviewer 2 suggests including additional studies published since 2024. I agree that both of these would improve the manuscript, however I will not make them a formal condition for acceptance if you acknowledge these issues as limitations instead.  Please note that this journal operates with transparent peer review. This means that if your submission is accepted for publication, the full peer review history of your article will publish online alongside your article. This includes reviewer comments, editor decision letters, and your author responses.  To revise your manuscript, log into https://mc.manuscriptcentral.com/oxfnsc and enter your Author Centre, where you will find your manuscript title listed under "Manuscripts with Decisions."  Under "Actions," click on "Create a Revision."  Your manuscript number has been appended to denote a revision.  You may also click the below link to start the revision process (or continue the process if you have already started your revision) for your manuscript. If you use the below link you will not be required to login to ScholarOne Manuscripts.  *** PLEASE NOTE: This is a two-step process. After clicking on the link, you will be directed to a webpage to confirm. ***  https://mc.manuscriptcentral.com/oxfnsc?URL_MASK=7b60c4cdc56745b099caed410a8e2c14  You will be unable to make your revisions on the originally submitted version of the manuscript.  Instead, revise your manuscript using a word processing program and save it on your computer.  Please also highlight the changes to your manuscript within the document by using the track changes mode in MS Word or by using bold or colored text.  <strong>REMINDER:</strong> if you have not already done so, when submitting your revised manuscript, please include a short alternative-text description under the figure legend for each figure. Alt-text is required to ensure the accessibility of our publications. Guidance is available on the journal’s Author Guidelines webpage and at <a href="https://academic.oup.com/pages/for-authors/journals/preparing-and-submitting-your-manuscript" target="_new">https://academic.oup.com/pages/for-authors/journals/preparing-and-submitting-your-manuscript</a>  Once the revised manuscript is prepared, you can upload it and submit it through your Author Centre.  When submitting your revised manuscript, you will be able to respond to the comments made by the reviewer(s) in the space provided.  You can use this space to document any changes you make to the original manuscript.  In order to expedite the processing of the revised manuscript, please be as specific as possible in your response to the reviewer(s).  IMPORTANT:  Your original files are available to you when you upload your revised manuscript.  Please delete any redundant files before completing the submission.  Because we are trying to facilitate timely publication of manuscripts submitted to the Oxford Open Neuroscience, your revised manuscript should be uploaded within the next 60 days from today date.  If it is not possible for you to submit your revision in a reasonable amount of time, we may have to consider your paper as a new submission.  Once again, thank you for submitting your manuscript to the Oxford Open Neuroscience and I look forward to receiving your revision.  Sincerely, Professor Sam Gilbert Senior Editor, Oxford Open Neuroscience sam.gilbert@ucl.ac.uk   Reviewer: 1  Comments to the Author This manuscript presents a coordinate-based activation likelihood estimation (ALE) meta-analysis comparing the neural correlates of explicit and implicit emotion regulation (ER). The authors synthesize neuroimaging data from 75 explicit ER experiments and 28 implicit ER experiments. The core finding is that explicit ER primarily recruits frontal and temporal regions associated with goal-directed behavior, whereas implicit ER relies more heavily on subcortical and limbic areas involved in emotional processing. Notably, the insula, inferior frontal gyrus, and extra-nuclear regions are identified as shared neural substrates. The study aims to move beyond the traditional focus on reappraisal and extinction by including a broader range of strategies. 1. The data analysis, while appropriate in its use of standard ALE methods, is presented in a rather direct and simplistic manner. A significant concern is the lack of validation regarding the stability and reliability of the observed clusters. Given the known impact of outlier experiments in coordinate-based meta-analysis, I strongly recommend that the authors supplement their results with a leave-one-out (LOO) sensitivity analysis. This procedure involves iteratively removing one experiment at a time and re-running the ALE to determine if the significant clusters remain consistent. Reporting which clusters survive this robustness check (and noting those that do not) would greatly strengthen the credibility of the conclusions. 2. Although the Introduction heavily emphasizes the transdiagnostic relevance of emotion regulation to psychopathology and mentions clinical interventions as a motivation, the Discussion fails to adequately bridge the gap between the neural findings and clinical practice. I suggest the authors expand the Discussion to specifically address the potential clinical implications of their findings.   Reviewer: 2  Comments to the Author The review addresses an important question on the neural basis of emotion regulation (i.e., the neural basis of explicitness vs implicitness) and the quantitative synthesis is valuable. The introduction is well written and critically sets the grounds for the review in a well thought out way. ALE methods are appropriate to answer the research question e.g., explicitness vs. implicitness. However, the current version requires revision to meet methodological standards for meta-analysis. Specifically, the literature searches have not been updated since 2024, despite relevant studies (and meta-analyses) published since then. Furthermore, the inclusion criteria for implicit emotion regulation studies requires clearer justification as it appears that some implicit emotion regulation paradigms may have been omitted from this review. Finally, some of the results of the ALE meta-analysis presented in Table 3 warrant discussion within the Discussion. Addressing these issues will strengthen the validity and comprehensiveness of the review. Please see the below for a detailed breakdown:   Highlights Correct typographical errors.   Abstract “We investigate whether explicit and implicit emotion regulation exist on a continuum of the explicitness of the emotion regulation goal neurally.”, This sentence should be clarified for readability as the meaning of the sentence does not clearly come through.  Some key points from the Introduction, Methods, Results, and Discussion should be included in the Abstract to improve the overall narrative. For example, the following conclusions should be considered for the Abstract: “The difference between implicit and explicit emotion regulation may indeed lie in the level of consciousness involved.”  The specific neural regions identified in the ALE meta-analysis should be presented in the Abstract for readers to get a complete picture.  Correct typographical errors (under Results).   Introduction Line 62-63: “which are methods used to modulate the expression of experience of emotions are referred to as emotion regulation strategies (Martínez-Priego et al., 2024).”, Improve wording for readability.  Line 66: “One biggest problem in both widely…”, Wording is contradictory (i.e., one and biggest) improve the whole sentence for readability.  “According to preliminary findings based on these limited focuses, implicit emotion regulation, with mixed degrees of controllability during regulation, recruits both prefrontal regions such as rostral anterior cingulate cortex (Fitzgerald et al., 2020), ventrolateral prefrontal cortex (Tupak et al., 2014) and regions that have rich connections to subcortical areas like insular cortices (Fullana et al., 2018). Explicit emotion regulation also recruits prefronto-parietal network and additionally deactivates limbic network (Buhle et al., 2014; Diekhof et al., 2011; Kohn et al., 2014; Ochsner et al., 2012).” Can the authors clarify whether this is within healthy subjects or individuals with mood and/or anxiety disorders.  Given the nature of this investigation into healthy subjects and individuals with mood and anxiety disorders, a concise summary of previous meta-analytical findings within explicit and implicit emotion regulation in both healthy and mood/anxiety disorder subjects should be included. For example, several previous ALE meta-analyses have investigated explicit and implicit emotion processing in mood and/or anxiety disorder populations. For example, see [Kohn, N., Eickhoff, S. B., Scheller, M., Laird, A. R., Fox, P. T., & Habel, U. (2014). Neural network of cognitive emotion regulation—an ALE meta-analysis and MACM analysis. Neuroimage, 87, 345-355], and [Dalton, S. D. P., Cooper, H., Jennings, B., & Cheeta, S. (2025). Neural correlates of implicit emotion regulation in mood and anxiety disorders: an fMRI meta-analytic review. Scientific Reports, 15(1), 19564.]  Following the above point, recent neuroimaging research on the brain regions associated with explicit and implicit emotion regulation should be revised to include recent work beyond 2024. Including such studies would strengthen the comprehensiveness and currency of the evidence.  The review’s aims should be more concise. Furthermore, some of this information belongs in the Methods rather than the Introduction e.g., “The most notable aspect of our study is the diverse range of strategies that we targeted, which differed along the continuum of the explicitness of emotion regulation goals. We included nine emotion regulation strategies in total that differ on the degree of explicitness of emotion regulation goal. In addition to widely studied strategies such as reappraisal and extinction, we also included strategies such as affect labelling (Torre & Lieberman, 2018) and the placebo effect (Ashar et al., 2017)…”, and “Since the neural studies of implicit emotion regulation used tasks that differ in the level of controllability, to yield in more studies in our analysis (Bruce et al., 2013; Pozzi et al., 2021; Tupak et al., 2014), we focused on the explicitness dimension only in the current analyses.”   Method A link should be included to the preregistered article on PROSPERO.  The authors have searched the previous 10 years of literature which is reasonable. However, since 2024 there have been additional neuroimaging studies and neuroimaging meta-analyses on explicit/implicit emotion regulation. Literature searches should be updated to include recent findings.  The authors should clarify how they handled different emotional stimuli e.g., positive or negative stimuli. If emotions were combined this should be stated.  A justification is needed as to why other implicit emotion regulation paradigms were not included in this review. Other tasks such as implicit emotional conflict adaptation and implicit cognitive reappraisal appear within the literature. The authors state they followed Braunstein et al.’s (2017) definitions. However, this should be clarified as potentially meaningful studies may have been omitted.  A quality assessment needs to be performed on the included studies to verify the quality of evidence/ risk of bias.   Results It should be made explicit in section “3.2 ALE analysis” that this is in healthy subjects.  Table 3 should clarify in healthy subjects.  The Results should include the specific neural regions that were identified in the ALE meta-analysis as stating, “Other clusters from the frontal lobe and parietal lobe were also reported.” does not provide sufficient detail for readers. Furthermore, previous meta-analysis has identified the medial frontal gyrus during implicit emotion regulation. This ALE meta-analysis has also identified this region in Table 3. All regions should be reported in text for readers to get a complete picture of the results. This should be considered for section 3.2.1., 3.2.2., 3.2.3., 3.2.4.  Results of a quality assessment should be included.   Discussion Update review of findings with respect to previous meta-analyses and other recent work since 2024.  The Discussion should be updated to include a brief discussion on the other regions also identified in the ALE meta-analysis e.g., the medial frontal gyrus during implicit emotion regulation.  If emotions were combined this should be noted as a limitation, as there could be meaningful differences in neural responses to positive versus negative stimuli.  If implicit emotion regulation paradigms were omitted due to strict criteria, this should be noted as a limitation as potentially relevant studies may have been missed.  Associate Editor Comments to the Author: (There are no comments.) |
| --- | --- |

**OXFNSC-2026-014.R1**

**28-Aug-2026**

Dear Dr. Lam,

It is a pleasure to accept your revised manuscript entitled "The Neural Basis of Explicit and Implicit Emotion Regulation: A Coordinate-Based Meta-Analysis" in its current form for publication in Oxford Open Neuroscience.  The comments of the reviewer(s) who reviewed your manuscript are included at the foot of this letter.

Please note that this journal operates with transparent peer review. This means that  the full peer review history of your article will publish online alongside your article. This includes reviewer comments, editor decision letters, and your author responses.

Next steps
You will receive an email from no-reply@scipris.com within roughly one week. This is your invitation to sign up for an account with SciPris, Oxford University Press’ author portal hosted by Aptara. You will need to create an account if you do not already hold one. Please register or log into your account and follow the online instructions which will guide you through signing your licence and paying the APC. The email and the portal have clearly signposted support options if you need any help during this process.

Please note that SciPris is a completely different system from ScholarOne, so your credentials to submit your manuscript here will not work there. Once you’ve created a SciPris account, you will be able to use it whenever you publish with Oxford Open Neuroscience or any OUP journal. Please note that OUP will only ever request payment for applicable fees be made via SciPris or to an OUP bank account. If you ever have concerns about the legitimacy of a request, please do not hesitate to contact a customer services agent via the SciPris portal or directly via oupsupport@scipris.com.

Thank you for your fine contribution.  On behalf of the Editors of the Oxford Open Neuroscience, we look forward to your continued contributions to the Journal.

Sincerely,
Professor Sam Gilbert
Senior Editor, Oxford Open Neuroscience
sam.gilbert@ucl.ac.uk


Reviewer: 1

Comments to the Author
Authors have addressed my comments well.

Reviewer: 2

Comments to the Author
The authors have suitably and appropriately actioned all comments and included relevant details within their manuscript where necessary (e.g., clarifying ER definitions, updating literature searches, clarifying neural regions within the Results, and including recent findings within the Introduction and Discussion). The manuscript has been strengthened and adheres to stringent meta-analytical protocol (e.g., performing a LOO sensitivity analysis). The manuscript is suitable at this stage for publication.

Associate Editor
Comments to the Author:
(There are no comments.)
